# Supplementary material for: Unveiling the mediating role of cultural trade and domestic identity in Chinese consumer engagement with foreign films and TV series
Source: PLoS One. 2024 Dec 16;19(12):e0314416. doi: 10.1371/journal.pone.0314416 (PMC11649089; doi:10.1371/journal.pone.0314416)
Supplement: S1 Appendix — (DOCX) [file pone.0314416.s002.docx]

**Appendix A: Questionnaire Questions and Results**

|  | **Question** |
| --- | --- |
| 1 | How often do you engage with foreign films and TV series? |
| 2 | How would you rate your emotional connection to foreign content? |
| 3 | How much do you spend monthly on foreign films and TV series? |
| 4 | Do you believe foreign films help in recognizing cultural diversity? |
| 5 | How much does foreign content influence your cultural practices? |
| 6 | Do you view foreign content as a means for intercultural exchange? |
| 7 | Does foreign content help you understand international sociocultural contexts? |
| 8 | Do you feel foreign films promote respect for other cultures? |
| 9 | How valuable do you find foreign films in terms of foreign language skill learning? |
| 10 | Do foreign films improve your cross-cultural sensitivity? |
| 11 | How much do you appreciate the artistic value in foreign content? |
| 12 | Do foreign films encourage you to have a broader view of different societies? |
| 13 | How do foreign films influence your personal growth and ethical views? |
| 14 | How important is intercultural economic exchange to you? |
| 15 | How do foreign films contribute to cultural diversity promotion? |
| 16 | How does cultural trade impact economic growth? |
| 17 | Do you consider foreign content as a factor in cultural industry development? |
| 18 | How important is cultural preservation and expansion in the context of global media? |
| 19 | How much pride do you have in your cultural heritage when engaging with foreign media? |
| 20 | Does foreign content help you understand your national culture better? |
| 21 | How does engaging with foreign content influence your perception of your culture globally? |
| 22 | What is your age? |
| 23 | What is your gender? |
| 24 | What is your highest education? |
| 25 | What is your occupation? |
| 26 | What is your annual income? |
