# Supplementary material for: Unveiling the mediating role of cultural trade and domestic identity in Chinese consumer engagement with foreign films and TV series
Source: PLoS One. 2024 Dec 16;19(12):e0314416. doi: 10.1371/journal.pone.0314416 (PMC11649089; doi:10.1371/journal.pone.0314416)
Supplement: Attachment — Submitted filename: Response to Reviewers.docx [file pone.0314416.s003.docx]

Response to ACADEMIC EDITOR Comments

The authors conducted a comprehensive review of the paper, submitting a version that could be accepted for publication after minor considerations:

**Point 1:** Include the nomenclature of each variable in Table 1

**Response 1:**

Thank you for your valuable feedback on our manuscript. We appreciate your insights and suggestions for improving the clarity and rigor of our work.

In response to your comment, we have included the nomenclature of each variable in Table 1 to ensure that all constructs and variables are clearly defined and easy to understand for readers. This addition aims to enhance the comprehensibility of our study and provide a more structured overview of the variables used in our analysis.

“In this study, the variables were measured meticulously to capture the intricate aspects of consumer behavior towards foreign films and TV series, ensuring that each dimension was analyzed with precision and depth. (see Table 1 and Appendix A)

**Table 1. Nomenclature of Dimensions and Constructs of Consumer Behavior and Cultural Perceptions in Foreign Films and TV Series**

| Dimension | Variable Symbol | Construct Description |
| --- | --- | --- |
| **Consumer Behavior (**$\mathbf{CB}_{\mathbf{0}}$**)** | $\mathrm{CB}_{P}$ | Engagement with foreign content, capturing frequency and enthusiasm for watching foreign films and TV series. |
|  | $\mathrm{CB}_{\mathrm{DE}}$ | Depth of commitment, indicating the emotional and time-related investment in foreign content. |
|  | $\mathrm{CB}_{\mathrm{AME}}$ | Economic and perceived value, reflecting financial expenditure and personal valuation of foreign entertainment. |
| **Cultural Value (**$\mathbf{CV}_{\mathbf{0}}$) | $\mathrm{CV}_{\mathrm{CP}}$ | Diversity recognition, highlighting the awareness and appreciation of varied cultures presented in media. |
|  | $\mathrm{CV}_{\mathrm{CC}}$ | Cultural learning tool, where foreign content is seen as a medium for understanding different cultural norms. |
|  | $\mathrm{CV}_{\mathrm{GCI}}$ | Global cultural exchange facilitator, promoting intercultural communication through content. |
|  | $\mathrm{CV}_{\mathrm{SHC}}$ | Sociopolitical context, using content to gain insight into international sociopolitical environments. |
|  | $\mathrm{CV}_{\mathrm{TUR}}$ | Understanding and respect, fostering empathy and respect for different cultures through foreign media. |
| Social and Practical Values ($\mathbf{SPV}_{\mathbf{0}}$) | $\mathrm{SPV}_{\mathrm{LSCE}}$ | Linguistic and cultural exchange value, assessing how content aids in cross-linguistic and cultural understanding. |
|  | $\mathrm{SPV}_{\mathrm{ICS}}$ | Cross-cultural communication skills, recognizing the role of media in enhancing interaction across cultures. |
|  | $\mathrm{SPV}_{\mathrm{AD}}$ | Artistic appreciation, reflecting the cultural and artistic enrichment from media consumption. |
|  | $\mathrm{SPV}_{\mathrm{EW}}$ | Societal understanding, encouraging a broadened perspective of other societies. |
|  | $\mathrm{SPV}_{\mathrm{PE}}$ | Personal growth and ethical development, assessing the influence on an individual's values and ethics. |
| Cultural Trade Identities ($\mathbf{CTI}_{\mathbf{0}}$) | $\mathrm{CTI}_{\mathrm{GCI}}$ | International dialogue and diversity, emphasizing the promotion of cultural diversity through global trade. |
|  | $\mathrm{CTI}_{\mathrm{CH}}$ | Cultural preservation, underlining the importance of maintaining and promoting national cultures through trade. |
|  | $\mathrm{CTI}_{\mathrm{EA}}$ | Economic amplification, stressing cultural trade's role in economic growth. |
|  | $\mathrm{CTI}_{\mathrm{ACI}}$ | Cultural industry development, focusing on expanding cultural industries through trade. |
|  | $\mathrm{CTI}_{\mathrm{ECC}}$ | Export sustainability, considering the sustainability of cultural trade practices. |
| **Domestic Cultural Identity (**$\mathbf{DCI}_{\mathbf{0}}$) | $\mathrm{DCI}_{\mathrm{CU}}$ | Unique cultural traits, highlighting distinctive national cultural features. |
|  | $\mathrm{DCI}_{\mathrm{CP}}$ | Cultural pride, reflecting individuals' pride in their national culture. |
|  | $\mathrm{DCI}_{\mathrm{UN}}$ | Cultural understanding, indicating deep personal knowledge of one’s own culture. |
|  | $\mathrm{DCI}_{\mathrm{CK}}$ | Cultural knowledge transmission, signifying the sharing and inheritance of cultural knowledge. |
|  | $\mathrm{DCI}_{\mathrm{CI}}$ | Cultural influence, focusing on how a nation’s culture impacts global awareness. |
| Consumer Characteristics ($\mathbf{CC}_{\mathbf{0}}$) | $\mathrm{CC}_{A}$ | Age as a factor, assessing how age influences content choices. |
|  | $\mathrm{CC}_{G}$ | Gender impact, examining how gender affects preferences and communication regarding media consumption. |
|  | $\mathrm{CC}_{E}$ | Education shaping values, exploring how educational background shapes personality, values, and preferences. |
|  | $\mathrm{CC}_{O}$ | Occupation influence, considering how occupation-related skills and behaviors affect media choices. |
|  | $\mathrm{CC}_{I}$ | Income effect, reflecting how income influences lifestyle and media consumption priorities. |

Please let us know if there are any other aspects that require further clarification or if you have additional suggestions.

**Point 2:** Include a table with the questions from the questionnaire in an appendix to the paper

**Response 2:**

Thank you very much for your constructive feedback on our manuscript. We sincerely appreciate your detailed review and recommendations for improving the quality of our work.

In response to your suggestion, we have now included a table with the questions from the questionnaire in an appendix to the paper. This addition aims to provide transparency and allow readers to fully understand the measures and constructs used in our study. We believe this enhancement will contribute significantly to the replicability and overall clarity of our research.

“**Appendix A: Questionnaire Questions and Results**

|  | **Question** |
| --- | --- |
| 1 | How often do you engage with foreign films and TV series? |
| 2 | How would you rate your emotional connection to foreign content? |
| 3 | How much do you spend monthly on foreign films and TV series? |
| 4 | Do you believe foreign films help in recognizing cultural diversity? |
| 5 | How much does foreign content influence your cultural practices? |
| 6 | Do you view foreign content as a means for intercultural exchange? |
| 7 | Does foreign content help you understand international sociocultural contexts? |
| 8 | Do you feel foreign films promote respect for other cultures? |
| 9 | How valuable do you find foreign films in terms of foreign language skill learning? |
| 10 | Do foreign films improve your cross-cultural sensitivity? |
| 11 | How much do you appreciate the artistic value in foreign content? |
| 12 | Do foreign films encourage you to have a broader view of different societies? |
| 13 | How do foreign films influence your personal growth and ethical views? |
| 14 | How important is intercultural economic exchange to you? |
| 15 | How do foreign films contribute to cultural diversity promotion? |
| 16 | How does cultural trade impact economic growth? |
| 17 | Do you consider foreign content as a factor in cultural industry development? |
| 18 | How important is cultural preservation and expansion in the context of global media? |
| 19 | How much pride do you have in your cultural heritage when engaging with foreign media? |
| 20 | Does foreign content help you understand your national culture better? |
| 21 | How does engaging with foreign content influence your perception of your culture globally? |
| 22 | What is your age? |
| 23 | What is your gender? |
| 24 | What is your highest education? |
| 25 | What is your occupation? |
| 26 | What is your annual income? |

”

Please feel free to let us know if there are any additional changes that you would recommend.

**Point 3:** Justify the choice of variables, age, gender and education, for the MGA analysis

**Response 3:**

Thank you very much for your insightful feedback and suggestions to improve our manuscript. We greatly appreciate your careful review.

Regarding your comment on justifying the choice of variables—age, gender, and education—for the MGA analysis, we have provided additional justification in the revised manuscript. Specifically, these variables were selected due to their well-established influence on consumer behavior in the context of foreign media consumption. Age affects openness to foreign cultural influences, as younger individuals are generally more exposed to global media through digital platforms. Gender influences media preferences, with different types of content resonating variably across male and female audiences. Education impacts cultural cognition, as individuals with higher educational attainment often show greater critical thinking abilities and a deeper appreciation for foreign cultural content. These factors make age, gender, and education crucial moderators in understanding the differentiated consumer engagement with foreign films and TV series.

“Age is a key determinant of consumer behavior, as individuals across different age groups display divergent value systems, beliefs, and priorities. These differences substantially affect their acceptance of foreign cultural content. Younger consumers are generally more open to diverse cultural influences, largely due to their exposure to globalization and their access to foreign cultures through digital platforms. Conversely, older consumers tend to favor content that aligns closely with their established cultural backgrounds. Hence, age serves as a significant factor in determining both the acceptance of and engagement with foreign films and television. The MGA in this study effectively highlights the pronounced distinctions across different age groups.

…

Gender influences individual preferences and communication styles, thereby affecting engagement with foreign audiovisual content. For instance, men and women may exhibit distinct preferences regarding media genres, with men often favoring action or sports-related content, while women may prefer drama or romance. Additionally, cultural roles and societal expectations linked to gender may moderate the degree of acceptance of foreign content. Thus, gender functions as a moderating variable that aids in comprehensively understanding the differences in the consumption of foreign cultural products.

…

Educational attainment significantly impacts an individual's value system, cultural cognition, and media consumption behavior. Higher education are generally correlated with greater critical thinking abilities and cultural appreciation, enabling individuals to understand foreign cultural content more profoundly, including its social and economic dimensions. Highly educated consumers tend to show a greater acceptance and appreciation for foreign films and television due to their capacity to derive cultural and intellectual value from such content.

…

Occupation determines not only an individual's economic capacity but also shapes personality traits and lifestyle preferences. The demand for and preference toward cultural content often vary according to occupational background. For example, full-time workers may prioritize entertainment and relaxation due to work pressures and time constraints, while their interest in cultural identity-related content might be weaker. Thus, occupation plays an indispensable role in influencing media consumption behaviors and serves as a critical variable in MGA.

…

Income level directly affects lifestyle, purchasing power, and individual priorities. High-income consumers typically possess greater social standing and broader access to cultural products, placing higher value on content quality and prestige, and often using foreign cultural products as a means of signaling social status. In contrast, lower-income consumers are more likely to focus on the utility and cost-effectiveness of cultural products. Therefore, income serves as a key variable in revealing differences in cultural consumption, particularly in terms of willingness to engage with and pay for foreign audiovisual content.

”

We hope this justification clarifies our approach, and we are open to further comments or suggestions you may have.

**Point 4:** Include after Figure 4, whether the hypothesis was accepted or rejected

**Response 4:**

Thank you for your constructive feedback and for pointing out areas for improvement. We truly value your input in enhancing the clarity and rigor of our manuscript.

In response to your suggestion, we have now included statements after Figure 4 indicating whether each hypothesis was accepted or rejected. Specifically, we have clearly stated the results of the hypothesis testing, based on the path coefficients and their statistical significance levels. This addition aims to improve the reader's understanding of the outcomes of our analysis and ensures that the conclusions drawn from the data are communicated transparently.

“The PLS-SEM analysis provides additional insights into Chinese consumer behavior in relation to foreign films and TV series (see Figure 4). Hypothesis 1 ($H_{1}$) receives partial support, with a strong positive relationship observed between perceived cultural values and consumer behavior towards foreign films and TV series ($\mathrm{CV}_{0}$ → $\mathrm{CB}_{0}$), with a path coefficient of 0.60. This suggests that cultural consumption allows consumers to express their identities and values, offering them a platform to explore and integrate diverse cultural paradigms. However, the relationship between perceived practical and social values and consumer behavior ($\mathrm{CV}_{0}$ → $\mathrm{CB}_{0}$) was found to be statistically insignificant. This could indicate that while cultural values are closely tied to personal identity and provide a meaningful emotional experience, practical and social values may not have the same influence on consumer behavior, as they do not offer the same depth of personal enrichment or emotional engagement.

|  | mean |
| --- | --- |
| $\mathrm{CV}_{0}$ → $\mathrm{CTI}_{0}$ → $\mathrm{CB}_{0}$ | 0.01 |
| $\mathrm{CV}_{0}$ → $\mathrm{DCI}_{0, UPN}$ → $\mathrm{CB}_{0}$ | 0.04* |
| $\mathrm{CV}_{0}$ → $\mathrm{DCI}_{0, IK}$ → $\mathrm{CB}_{0}$ | 0.03* |
| $\mathrm{SPV}_{0}$ → $\mathrm{CTI}_{0}$ → $\mathrm{CB}_{0}$ | 0.02 |
| $\mathrm{SPV}_{0}$ → $\mathrm{DCI}_{0, UPN}$ → $\mathrm{CB}_{0}$ | 0.13*** |
| $\mathrm{SPV}_{0}$ → $\mathrm{DCI}_{0,IK}$ → $\mathrm{CB}_{0}$ | 0.01 |

**Fig 4. The results of PLS-SEM analysis.**

Note: *** p < 1%, ** p < 5%, * p < 10%.

Hypothesis 2 ($H_{2}$) is substantiated to a degree, as evidenced by the statistically significant positive mediating effects of the emotional and subjective dimensions of domestic cultural identity on both cultural values and practical and social values in shaping consumer behavior towards foreign films and TV series. Specifically, the indirect effects in the pathways from cultural values to domestic cultural identity to consumer behavior, and from practical and social values to domestic cultural identity to consumer behavior ($\mathrm{CV}_{0}$ → $\mathrm{DCI}_{0, UPN}$ → $\mathrm{CB}_{0}$ and $\mathrm{SPV}_{0}$ → $\mathrm{DCI}_{0, UPN}$ → $\mathrm{CB}_{0}$), are measured at 0.04 and 0.13, respectively. Furthermore, a notable positive indirect effect is observed in the pathway from cultural values to consumer behavior through the knowledge-based dimension of domestic cultural identity ($\mathrm{CV}_{0}$ → $\mathrm{DCI}_{0,IK}$ → $\mathrm{CB}_{0}$), quantified at 0.03.”

We hope this addresses your comment effectively, and we are more than willing to make further revisions if necessary.

**Point 5:** Consider a discussion section and one just for the conclusion

**Response 5:**

Thank you very much for your valuable feedback and thoughtful suggestions on our manuscript. We are grateful for your insights, which have greatly contributed to improving our work.

In response to your suggestion, we have now restructured the manuscript to include distinct sections for the discussion and the conclusion. The discussion section focuses on interpreting the findings in light of existing literature, exploring the implications, and providing a critical analysis of the results. The conclusion section succinctly summarizes the key findings, outlines the contributions of the study, and provides suggestions for future research. This separation aims to enhance the clarity and coherence of our argument, ensuring that readers can easily differentiate between the interpretation of findings and the final summarizing statements.

We hope that these modifications meet your expectations, and we are open to any further recommendations you may have.

**Point 6:** Make clearer in the conclusions what the contributions of the study are to theoretical and practical aspects, and what the limitations and recommendations for future studies are.

**Response 6:**

Thank you for your insightful feedback and for helping us improve the clarity of our manuscript. We greatly value your suggestions.

In response to your comment, we have revised the conclusions section to clearly delineate the theoretical and practical contributions of the study. Specifically, we have highlighted how our research advances theoretical understanding by integrating cultural hybridization theory with consumer behavior models, thus providing insights into the mediating and moderating effects of cultural identity on media consumption. Practically, we have elaborated on how our findings can guide marketers and content creators in effectively targeting diverse demographic segments in China by tailoring culturally resonant media content.

We have also included a more explicit discussion of the study's limitations, such as the reliance on self-reported data and the focus on a specific cultural context, which may affect generalizability. Additionally, we have provided recommendations for future research, including exploring other demographic variables, using longitudinal data, and extending the study to different cultural settings to enhance external validity.

“**Conclusions**

The research investigates the influence of foreign films and TV series on the behavior of Chinese consumers, focusing on how demographic factors, such as age, gender, education, occupation, and income, influence consumer engagement with these media. The study is set against the backdrop of globalization and the proliferation of streaming platforms, which have facilitated increased cultural exchange and access to foreign media in China. The research also explores the role of cultural trade and domestic cultural identity in shaping consumer preferences.

The primary objective of this research is to clarify the causal relationships between consumers' perceptions of the cultural, utilitarian, and societal values inherent in foreign films and TV series and their subsequent consumption patterns. The study aims to uncover the mediating roles played by cultural trade and domestic cultural identities, and to assess the moderating effects of individual characteristics on these relationships.

The research employs a mixed-methods approach, starting with a comprehensive literature review followed by a questionnaire survey to collect primary data from 786 respondents. Descriptive statistical analyses are used to analyze initial data, which are further refined using Partial Least Squares Structural Equation Modeling (PLS-SEM) to establish complex interrelationships among variables. Multi-group analysis (MGA) is also conducted to examine the moderating effects of demographic factors, including age, gender, education, occupation, and income, on consumer behavior.

The findings reveal that cultural values significantly influence consumer engagement with foreign films and TV series, whereas practical and social values do not exhibit the same level of impact. Domestic cultural identity serves as a significant mediator between cultural values and consumer behavior, whereas cultural trade identity does not have a notable mediating effect. Additionally, consumer demographics, such as age, gender, education, occupation, and income, significantly moderate the relationships between cultural values, cultural identity, and consumer engagement.

This study builds on existing consumer behavior theories by emphasizing the role of cultural values, domestic cultural identity, and social identity in shaping media consumption. It expands on Hofstede's [17] Cultural Dimensions theory and Zhao and Belk's [65] consumer behavior insights, showing that domestic cultural identity has a more substantial influence than cultural trade identity. The finding also challenges the assumptions that global cultural trade would significantly influence consumer patterns, instead highlighting the enduring power of national and cultural pride in consumers’ engagement with global media content. This contributes to a broader framework of consumer behavior by highlighting the significance of emotional factors and personal identity (rather than merely social or practical considerations) in driving media consumption, particularly within a globalized media landscape.

For marketers and content creators, the study underscores the need for cultural sensitivity when producing and promoting foreign films and TV series. It shows that aligning content with domestic cultural identity, particularly in environments where cultural and personal values are closely linked, is crucial for engagement. Furthermore, demographic factors such as age, education, and income play critical roles in moderating consumer behavior, highlighting the necessity for media companies to develop more targeted and personalized campaigns. Overall, these findings demonstrate the importance of incorporating both cultural and demographic specificity into marketing strategies of foreign films and TV series.

The study contributes to the theoretical understanding of consumer behavior by integrating cultural hybridization theory, uses and gratifications theory, and theories of planned behavior to explain the complex dynamics of consumer engagement with foreign films and TV series. It advances the literature by highlighting the critical mediating role of domestic cultural identity and demonstrating the differential effects of demographic moderators on consumer behavior.

From a practical perspective, the study provides valuable insights for marketers and content creators aiming to penetrate the Chinese media market. It emphasizes the importance of cultural alignment and sensitivity in developing content that resonates with Chinese audiences. The findings suggest that producers and marketers should prioritize culturally resonant themes and understand the unique preferences of different demographic groups to effectively engage the target audience and enhance market success.

The study's reliance on convenience sampling presents a key limitation, as it does not fully capture the population's diversity. This method can lead to demographic biases and limit the generalizability of the results. Additionally, the use of self-reported data introduces the possibility of response bias, where respondents may offer socially desirable answers rather than accurate reflections of their preferences. The study’s cross-sectional design, which examines behavior at a single point in time, also limits the ability to assess behavioral changes over time or to establish causality between variables. Lastly, the study’s focus on Chinese consumers restricts the global applicability of the findings, as cultural dynamics may differ in other regions.

To overcome these limitations, future studies should adopt a more diverse sampling method, such as stratified random sampling, to ensure broader representation. Longitudinal studies are recommended to track changes in consumer behavior over time, allowing for a more comprehensive understanding of how cultural values, cultural trade identity, and domestic cultural identity influence media consumption. Expanding the research to include respondents from various cultural backgrounds would also enhance the global applicability of the findings. Moreover, qualitative methods such as interviews and focus groups can provide deeper insights into the motivations and subjective experiences driving consumer engagement with foreign films and TV series. Lastly, the increasing role of AI and digital technologies in shaping consumer behavior, particularly in the context of AI-generated content in foreign films and TV series, offers a promising avenue for future research. Understanding the long-term impacts of these technological advancements on cultural consumption is essential for navigating the evolving media landscape.”

We hope that these revisions sufficiently address your feedback, and we are happy to make further improvements if necessary.
